# Risk factors for post-endoscopic retrograde cholangiopancreatography cholangitis in patients with hepatic alveolar echinococcosis—an observational study

**DOI:** 10.1080/07853890.2022.2091792

**Published:** 2022-07-06

**Authors:** Fei Du, Wenhao Yu, Zhixin Wang, Zhi Xie, Li Ren

**Affiliations:** Department of Hepatic-Biliary-Pancreatic Surgery, Affiliated Hospital of Qinghai University, Xining, China

**Keywords:** Endoscopic retrograde cholangiopancreatography, cholangitis, alveolar echinococcosis, risk factors

## Abstract

**Background:**

Hepatic alveolar echinococcosis (HAE) is considered to be one of the most deadly chronic parasitic diseases in the world. We have shown that the incidence of cholangitis in patients who underwent endoscopic retrograde cholangiopancreatography (ERCP) was increased significantly. On this finding, we carried out, a preliminary study on the risk factors for cholangitis after ERCP.

**Aims:**

To retrospectively detect the risk factors for post-ERCP cholangitis in patients with biliary tract affected by HAE.

**Methods:**

The study included data from 51 cases of AE who had undergone therapeutic ERCP between January 2015 and December 2019. Demographic and treatment data were extracted from the medical records, and the association between potential risk factors and the development of post-ERCP cholangitis was evaluated using a collected database.

**Results:**

There were five cases of mild cholangitis after ERCP (Tokyo criteria), and no moderate or severe cholangitis occurred. The incidence rate of cholangitis after ERCP was 9.8%. Univariate analysis showed hilar bile duct stenosis (*p* = .016), endoscopic retrograde biliary drainage (*p* = .007), a stent diameter ≥8.5 Fr (*p* = .000) and single stent implantation (*p* = .010) were risk factors for post -ERCP cholangitis. All cases of cholangitis improved under conservative treatment.

**Conclusion:**

Patients with hilar bile duct compression or endoscopic retrograde biliary drainage appeared to be more likely to develop post-ERCP cholangitis. The number and diameter of biliary stents may influence post-ERCP cholangitis. Sample size and clinical heterogeneity are two insurmountable difficulties, and a larger sample size needs to be collected to verify the risk factors for screening.

**KEY MESSAGES**
- Many studies reported the post-ERCP complications in patients with hepatic alveolar echinococcosis and found that the incidence of post-ERCP cholangitis was significantly high. Therefore, we conducted a preliminary study on the risk factors of postoperative cholangitis in patients who underwent ERCP.
- The incidence rate of cholangitis after ERCP was 9.8%. We found that hilar bile duct stenosis, and endoscopic retrograde biliary drainage, were risk factors for cholangitis, and stent diameter and the number of stent implantation may influence the incidence rate of cholangitis after ERCP.
- Sample size and clinical heterogeneity are two insurmountable difficulties, and a larger sample size needs to be collected to verify the risk factors of screening.

## Introduction

Hepatic alveolar echinococcosis (HAE) is considered to be one of the most deadly chronic parasitic diseases in the world, posing a serious threat to life and health [1–4]. In humans, AE larvae develop almost exclusively in the liver, leading to slowly progressive, life-threatening tumor-like growth [5,6]. Without treatment, 90–100% of AE patients die 10–15 years after diagnosis [7]. China is estimated to account for more than 90% of AE cases worldwide [8,9]. In particular, Qinghai and the Tibet autonomous region have incurred considerable human costs due to a lack of therapeutic treatment programs [10].

To date, the combination of surgery and administration of albendazole for one year is considered the primary radical treatment for patients diagnosed clinically with AE [11]. However, because early AE patients have no clinical symptoms, the majority of patients are already in the advanced stage of the disease by the time they are diagnosed and seek treatment [12]. Percutaneous intrahepatic bile duct drainage or endoscopic retrograde cholangiopancreatography (ERCP) should be performed in patients with a lost surgical opportunity or postoperative biliary complications to improve their quality of life. There are many studies on the efficacy of endoscopic treatment for biliary tract complications in AE patients, with the prevention of complications after ERCP also forming an important part of this research. Although severe complications can lead to decreased quality of life, increased hospitalization costs, and even death, there are no relevant reports on risk factors for complications after ERCP in AE patients requiring endoscopic intervention.

Graeter et al. [13], Ren et al. [14], and a European multi-center retrospective analysis [15] reported the post-ERCP complications and found that the incidence of post-ERCP cholangitis was significantly higher than for other diseases. Therefore, we conducted a preliminary study on the risk factors of cholangitis in patients who underwent ERCP.

## Materials and methods

### Patients

We retrospectively reviewed the endoscopic database of our hospital to identify patients with HAE who received ERCP between January 2015 and December 2019. This resulted in 51 cases that occurred in 45 patients of AE being enrolled in this study. The diagnosis of AE was based on the Epidemiological history of the infected area, pathological biopsy, ultrasound, computed tomography, magnetic resonance imaging, and liver function tests. The World Health Organization established the classification and diagnostic criteria for AE [16], with the diagnosis of the biliary system condition combined with computed tomography, magnetic resonance imaging, and ERCP. All the procedures were performed with the written consent of the patient. The study was approved by our hospital ethics committee (Number P-SL-2019042).

### Examination and indications

All examinations are performed by an experienced chief surgeon (over 20 years in endoscopy and over 600 ERCP cases per year). Side-viewing duodenoscopes (Pentax EPK-i5000; HOYA Corporation, Nishi-shiniuku, Shinjuku-ku Tokyo, Japan) were used to examine all the patients. All patients were treated intraoperatively with anti-inflammatory therapy (gentamicin added to the contrast medium) and postoperatively (using ceftriaxone). This study included the following inclusion criteria: (a) patients with a malignant obstruction caused by compression of the hilar bile duct; (b) patients with a biliary fistula who required endoscopic intervention; (c) patients who required endoscopic nasobiliary drainage to provide radiography before hepatectomy; and (d) patients with a hilar bile duct stricture after hepatectomy. Exclusion criteria: (a) patients with jaundice due to liver injury caused by AE but without biliary obstruction; (b) patients with combined hepatic cystic echinococcosis.

### ERCP process

All patients were placed in the left-sided prone position, general anaesthesia with propofol was administered intravenously, and intraoperative oxygen and cardiac monitoring to monitor the patients' vital signs. All patients were intubated with conventional guidewire guidance. When intubation was difficult, double guidewire intubation and pre-cut sphincterotomy were used to assist intubation. After insertion of the guidewire, a contrast catheter was inserted to inject ultravist for imaging to assess the status of the patient's common bile duct and intrahepatic bile duct. In patients with extrahepatic biliary strictures, guidewire-guided balloon dilation followed by biliary stent placement was performed. In the case of intrahepatic biliary strictures, one or more plastic stents are placed for drainage as appropriate, depending on the guidelines and the physician's experience. In case of combined common bile duct stones, a reticular basket or balloon catheter was used to remove the stones.

### Covariates

The following independent variables were assessed as possible risk factors for post-ERCP cholangitis: choledocholithiasis, biliary fistula, liver surgery (palliative and radical surgery), choledochal stricture or dilatation, hilar stricture, gallbladder status, Alkaline phosphatase, total bilirubin, Child-Pugh grade, endoscopic sphincterotomy, endoscopic retrograde biliary drainage, endoscopic nasobiliary drainage, duodenal papillary balloon dilatation, percutaneous transhepatic cholangial drainage and the diameter and number of stents.

### Assessments

The primary endpoint of the analysis was the incidence of post-ERCP cholangitis. It is diagnosed according to the standard dictionary of endoscopic complications [17] issued by the American Society for Gastrointestinal Endoscopy (ASGE). Post-ERCP cholangitis was defined as a postoperative biliary fever (body temperature >38 °C), no preoperative fever, acute cholestasis with no cholecystitis, and other possible infections. The post-surgical biliary fistula was defined as either: fluid with an increased bilirubin concentration in the abdominal drain or the intra-abdominal fluid on or after postoperative day 3; the need for radiologic intervention; grade B bile leakage requiring a change in the patient’s clinical management but manageable without relaparotomy; or a grade A bile leakage lasting for >1 week [18]. The diagnosis of a non-surgical biliary fistula was based on biochemical tests, an imaging examination, or ERCP.

### Statistical methods

The data were analyzed using Statistical Package for Social Science (SPSS Inc., Chicago, IL, USA) version 18.0. The data were expressed as mean ± standard deviation (*SD*) for continuous variables, while categorical variables were expressed as the number of cases (percentage). Differences between the two groups were analyzed using Student’s *t*-test or the Mann–Whitney *U* test for continuous variables and the *χ*^2^ test or Fisher's exact test for categorical data. *p*-Values <.05 were considered statistically significant.

## Results

### Demographic characteristics

**Figure 1.**
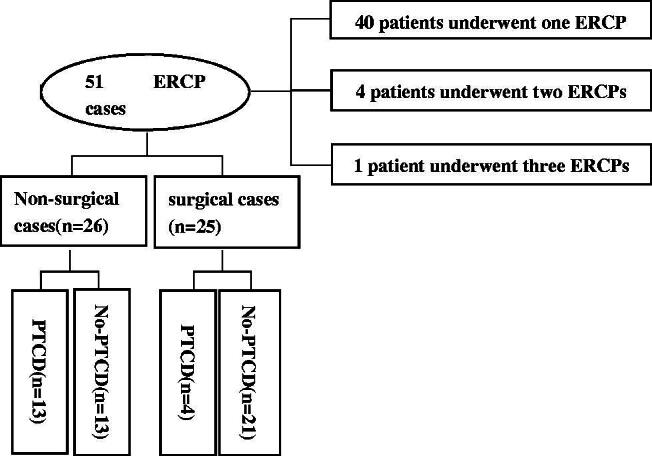
Flow chart.

Fifty-one ERCP cases were included, of which 40 patients underwent one ERCP, and five patients underwent multiple ERCPs (four patients two times and one patient three times) (Figure 1). The mean age of the patients was 38.27 (SD,12.77) years, with a range of 14–67 years. Of the 45 patients, 21 were males (46.7%) and 24 were females (53.3%) (Table 1).

**Table 1.** Patient characteristics.

Demographic characteristics	
Gender (male *vs.* female)	21:24
Age (year)	43.17 (*SD*: 12.28)
Clinical symptoms (*n*)	
Epigastric pain	29
Jaundice	10
Epigastric pain with jaundice	5
Other symptoms	7
Surgical intervention	25 (49.0%)
Radical hepatectomy	18
RH/LH/others	8/5/5
Palliative Operation	7
RHLCD/EHEL	2/5
Non-operative intervention	26 (51%)
Lesion stages P4/P3/P2	4/19/3
Involved parts R/L/R + L	20/4/2
Diameter of the lesion	13.2 cm (*SD* 3.8)
Preoperative and intraoperative operations	
PTCD	17 (33.3%)
EST	18 (35.3%)
EPBD	13 (25.5%)
ERBD	20 (39.2%)
ENBD	24 (47.1%)

RH: right hemihepatectomy; LH: left hemihepatectomy; RHLCD: right lobe of liver hydatid liquefaction cavity drainage; EHEL: excision of hepatic echinococcosis lesion; R: right liver; L: left liver; EST: endoscopic sphincterotomy; ERBD: endoscopic retrograde biliary drainage; ENBD: endoscopic nasobiliary drainage; EPBD: duodenal papillary balloon dilatation; PTCD: percutaneous transhepatic cholangial drainage.

### Basic clinical characteristics

#### Clinical symptoms

As shown in Table 1, the main clinical symptoms were epigastric pain in 29 cases (56.9%), jaundice in 10 cases (19.6%), epigastric pain with jaundice in five cases (9.8%), and seven cases with other symptoms (13.7%).

#### Diagnostic grading, location, and size of the occupation

In the retrospective ERCP cases, 26 cases were not treated by liver surgery, including four cases in P4 stage, 19 in P3, and three in P2. The maximum cross-sectional diameter of the lesion was 13.2 cm (*SD*, 3.8), with eight patients undergoing surgery within one week. The purpose of ERCP was to provide better cholangiography during the operation. Of the cases that did not require surgery, the lesions mainly involved the left lobe of the liver in four cases, the right lobe in 20 cases, and both lobes in two cases (Table 1).

#### Surgery for AE before ERCP

In the retrospective ERCP cases, 25 were diagnosed with AE and underwent radical hepatectomy or palliative surgery 18 cases underwent a radical hepatectomy (eight right hemihepatectomy, five left hemihepatectomy, two right hemihepatectomy plus caudate lobectomy, one S8-segment resection, one S4-segment resection with microwave ablation of the caudate lobe, and one total isolated autologous liver transplantation); and seven cases were treated by palliative lesion resection (two with drainage of the hydatid liquefaction cavity in the right lobe of the liver and five who had a resection of hydatid lesion (Table 1).

#### Preoperative and intraoperative operation

Of the 51 cases, 17 received percutaneous transhepatic cholangial drainage to relieve obstructive jaundice before ERCP. Eighteen cases were treated by endoscopic sphincterotomy, 13 by duodenal papillary balloon dilatation, 20 by endoscopic retrograde biliary drainage, and 24 by endoscopic nasobiliary drainage (Table 1).

#### Post-ERCP cholangitis and univariate analysis

There were five cases of mild cholangitis after ERCP (Tokyo criteria [19]), and no moderate or severe cholangitis occurred.

The patients with the complications described above recovered quickly under medication and did not develop any further serious complications. As shown in Table 2, the incidence rate of cholangitis after ERCP in the study was 9.8%. Univariate analysis showed that stenosis (*p* = .016), endoscopic retrograde biliary drainage (*p* = .007), a stent diameter ≥8.5 Fr (*p* = .000), and single stent implantation (*p* = .010) were risk factors for cholangitis (Table 2).

**Table 2.** Univariate analysis of post-ERCP cholangitis.

	Cholangitis group (*n* = 5)	Non-cholangitis group (*n* = 46)	*p*-Value
Choledocholithiasis	2	8	.250^a^
Biliary fistula	0	12	.323^a^
No liver surgery	3	23	.999^a^
Oral albendazole	2	22	.999^a^
Normal common bile duct	2	25	.826^a^
Common bile duct stenosis	1	12	
Choledochectasis	2	9	
Hilar bile duct stenosis	5	2	.016^a^
Normal gallbladder	2	25	.826^a^
Gall bladder removal	2	12	
Chronic cholecystitis	1	9	
Alkaline phosphatase (U/L)	707.5 (67, 33,260)	205.0 (7.3, 487)	.066
Total bilirubin (umol/L)	23.2 (15.0, 238.0)	107.5 (4.3, 543.0)	.569
Child-Pugh levels A	2	9	.159^a^
2003 B/C	1/2	28/9	
EST	3	15	.331^a^
ERBD	5	15	.007^a^
Stent diameter			.000^a^
≥8.5 Fr	5	4	
<8.5 Fr	0	11	.000^b^
No stent	0	31	.008^b^
Stent implantation			.008^a^
No stent implantation	0	31	
Single stent implantation	4	12	.010^c^
Multiple stent implantation	1	3	.114^c^
ENBD	1	23	.354^a^
EPBD	2	11	.591^a^
PTCD	3	14	.318^a^

EST: endoscopic sphincterotomy; ERBD: endoscopic retrograde biliary drainage; ENBD: endoscopic nasobiliary drainage; EPBD: duodenal papillary balloon dilatation; PTCD: percutaneous transhepatic cholangial drainage.

^a^*p*-Value obtained from Fisher exact test.

^b^Compared to Stent diameter ≥8.5 Fr.

^c^Compared to No stent implantation.

## Discussion

The incidence of cholangitis after ERCP is about 0.5–5% [20] and has a high mortality rate of about 4.5–8% [21,22]. In most cases of cholangitis, it is not clear how bacteria enter the obstructed bile duct. Several studies have shown a direct relationship between the development of bacteraemia or endotoxemia and the pressure within the biliary system [23–26]. In addition, the reverse invasion of ERCP can lead rapidly to bacterial translocation, colonization, and cholangitis [27]. An obstructed biliary tract also promotes bacterial translocation to normally sterile sites [28]. When biliary obstruction leads to elevated biliary pressure, bacteria and bacterial products can retrograde from the bile and leak into the body circulation, leading to clinical manifestations of sepsis and cholangitis.

Studies on risk factors for cholangitis after ERCP are scarce and results are variable. The different results may be due to the small number of patients and the different populations and methods used in the studies. This study showed that although our patients with AE were routinely administered antibiotics during and one day after ERCP, the incidence of post-ERCP cholangitis remained high. In our opinion, patients who undergo palliative or radical hepatectomy should be in better physical condition than those not receiving surgery, although surgical intervention before ERCP was clearly not associated with a high incidence of post-ERCP cholangitis in this study.

Univariate analysis showed that hilar bile duct stricture and endoscopic retrograde biliary drainage were risk factors for the development of post-ERCP cholangitis. The hilar bile duct strictures in our patients were divided mainly into two types either caused by AE invasion or as a post-hepatectomy complication. In the AE invasion group of patients, the anatomical complexity and significant anatomical deformities of the hilar bile duct [29] may have led to longer ERCP times, with the growth of the lesion tending to cause stent occlusion [29], and a higher risk of post-ERCP cholangitis. AE has aggressive growth characteristics similar to tumours, resulting in cholangitis similar to malignant hilar biliary obstruction after ERCP. In t post-hepatectomy complication group of patients, post-surgical hilar bile duct strictures were a more difficult technical procedure, resulting in inadequate drainage. In this regard, contrast media has been reported to cause post-ERCP cholangitis [30].


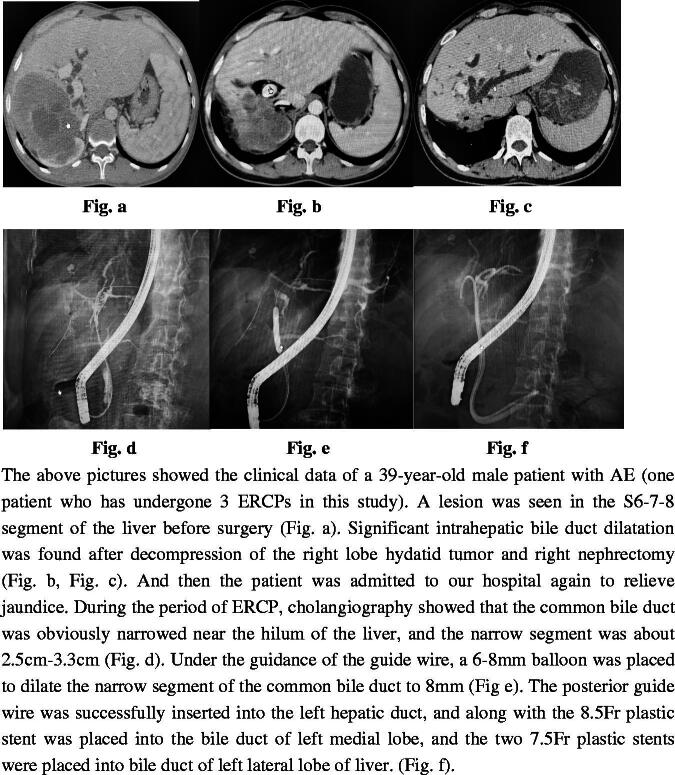


We also found that endoscopic retrograde biliary drainage was another risk factor for post-ERCP cholangitis. The diameter of the biliary stents we implanted was either 6.5, 7.5, 8.5, or 10 Fr, based mainly on the experience of the endoscopic surgeon during the operation. However, we found that all patients with post-ERCP cholangitis had received single stent implantation with stents larger than 8.5 Fr in diameter, which may have caused post-ERCP biliary dysfunction and bile reflux to the liver. In addition, some studies have suggested that biliary stent implantation is associated with a high incidence of post-ERCP cholangitis [31–33], which is consistent with the finding of our study. Gianfranco et al.'s research support this view. The study [34] shows that there is microbial colonization in polyethylene stents, and polyethylene plastic stents are used in our cases. Since we do not have data on stent culture to confirm our hypothesis about stent infection, further research is needed. Furthermore, the bacterial migration caused by the loss of the enterobiliary barrier after stent implantation may be an important reason for the increase in bacterial catheter colonization and multi-bacterial culture [25].

To prevent post-ERCP cholangitis it may be prudent to replace endoscopic retrograde biliary drainage with endoscopic nasobiliary drainage. However, this will worsen the patient's quality of life, and the nasobiliary duct is more likely to become blocked, which represents a dilemma for surgeons [35]. Replacement with a self-expandable metallic stent (SEMS) in the hilar bile duct does not appear feasible because it is more likely to lead to contra-lateral bile duct occlusion [36]. We propose to implant multiple, removable, small-diameter plastic stents to achieve satisfactory drainage while avoiding stent displacement and preventing post-ERCP cholangitis.

We consider that our preliminary report may provide a valuable reference for understanding the incidence of post-ERCP cholangitis and ultimately helping doctors to prevent cholangitis. In addition, when doctors perform ERCP, it is important to give detailed informed consent to the treated patients and their families, including the patient's risk of developing cholangitis.

## Shortcoming

Although the study had a relatively large sample size for a rare disease, such as AE, the sample size was insufficient for a valid multivariate analysis. Therefore, this study only used simple statistical analysis methods to identify possible risk factors, which may have adversely affected the authenticity of the results. We still think this is a very interesting finding. We plan to increase the sample size for reanalysis in the future. In addition, this study was a single-center experiment, so it may not represent the ethnic and regional differences involved in the development of AE.

## Conclusion

Patients with hilar bile duct compression or endoscopic retrograde biliary drainage appeared to be more likely to develop post-ERCP cholangitis. The number and diameter of biliary stents may influence cholangitis. Sample size and clinical heterogeneity are two insurmountable difficulties, and a larger sample size needs to be collected to verify the risk factors of screening.

## Data Availability

The data that support the findings of this study are available from the corresponding author, [L.R.], upon reasonable request.
